# Measurement of *Plasmodium falciparum* transmission intensity using serological cohort data from Indonesian schoolchildren

**DOI:** 10.1186/1475-2875-12-21

**Published:** 2013-01-17

**Authors:** Michael T Bretscher, Supargiyono Supargiyono, Mahardika A Wijayanti, Dian Nugraheni, Anis N Widyastuti, Neil F Lobo, William A Hawley, Jackie Cook, Chris J Drakeley

**Affiliations:** 1 Department of Immunology & Infection, London School of Hygiene and Tropical Medicine, London W1CE 7HT, UK; 2 Center for Tropical Medicine Faculty of Medicine, Gadjah Mada University, Yogyakarta, Indonesia; 3 Eck Institute for Global Health, University of Notre Dame, Notre Dame, IN 46556, USA; 4 UNICEF, Jakarta, Indonesia; 5 Department of Medicine Solna Malaria Research Unit, Karolinska Institutet, Stockholm, Sweden

**Keywords:** Malaria, *Plasmodium falciparum*, Serology, Epidemiology, Cohort study, Force of infection, Measuring transmission intensity, Antibodies, Elimination, Longitudinal data

## Abstract

**Background:**

As malaria transmission intensity approaches zero, measuring it becomes progressively more difficult and inefficient because parasite-positive individuals are hard to detect. This situation may arise shortly before achieving local elimination, or during surveillance post-elimination to prevent reintroduction. Antibody responses against the parasite last longer than the infections themselves. This “footprint” of infection may thus be used for assessing transmission intensity. A statistical approach is presented for measuring the seroconversion rate (SCR), a correlate of the force of infection, from individual-level longitudinal data on antibody titres in an area of low *Plasmodium falciparum* transmission.

**Methods:**

Blood samples were collected from 160 Indonesian schoolchildren every month for six months. Titres of antibodies against AMA-1 and MSP-1_19_ antigens of *P. falciparum* were measured using ELISA. The distribution of antibody titres among seronegative and -positive individuals, respectively, was estimated by comparing the titres from the study data (a mixture of both seropositive and -negative individuals) with titres from a (unexposed) negative control group of Indonesian individuals. Two Markov-Chain models for the transition of individuals between serological states were fitted to individual anti-PfAMA-1 or anti-PfMSP-1 titre time series using Bayesian Markov-Chain-Monte-Carlo (MCMC). This yielded estimates of SCR as well as of the duration of seropositivity.

**Results:**

A posterior median SCR of 0.02 (Pf AMA-1) and 0.09 (PfMSP-1) person^-1^ year^-1^ was estimated, with credible intervals ranging from 1E-4 to 0.2 person^-1^ year^-1^. This level of transmission intensity is at the lower range of what can reliably be measured with the present study size. A Bayesian test for seroconversion of an individual between two observations is presented and used to identify the subjects who have most likely experienced an infection. Furthermore, the theoretical limits of measuring transmission intensity, and how these depend on duration and size of a study as well as on transmission intensity itself, is illustrated.

**Conclusions:**

This analysis shows that it is possible to measure SCR's from individual-level longitudinal data on antibody titres. In addition, individual seroconversion events can be identified, which can be useful in assessing interruption of transmission. Analyses of further serological datasets using the present method are required to improve and validate it. This includes measurement of the duration of antibody responses, how it depends on host age or cumulative exposure, or on the particular antigen used.

## Background

Human malaria caused by the *Plasmodium falciparum* parasite is a public health priority in many sub-tropical and tropical areas. Intervention programs aiming at either reduction of malaria transmission intensity to minimize morbidity and mortality (“control”) or aiming at local interruption of parasite transmission (“elimination”) require continuous monitoring of transmission levels [1].

A natural measure of transmission intensity is the force of infection parameter (FOI), which corresponds to the number of infections acquired per person per year. More familiar is the entomological inoculation rate (EIR, number of infectious mosquito bites per person and year). EIR values tend to be higher than values of FOI, because not all mosquito bites result in an infection. Parasite prevalence (the proportion of the population which is parasite positive) increases monotonically with EIR but saturates at high transmission levels. At medium to high FOI these quantities may be directly measured from data on presence of the parasite: the number of sporozoite-positive mosquitoes which bite a person per night can be used to estimate EIR, and diagnostic methods for detection of parasites in the human population – such as microscopy, rapid diagnostic tests (RDT) or polymerase chain reaction (PCR) – yield estimates of parasite prevalence. At very low transmission intensities, however, detection of the parasite itself becomes very inefficient: enormous study populations would be required in order to find enough parasite-positive individuals. Similarly, large numbers of mosquitoes would need to be captured in order to gain reliable EIR estimates [2]. The presence of antibodies against the parasite, in contrast, has classically been used to measure transmission at low levels, and correlates well with EIR [3,4]. How long this “footprint” of an infection persists is debated, but even in the worst case antibodies are present in blood longer than the parasite itself [5]. The seroconversion rate (SCR, the number of individuals which become seropositive per person and year) thus constitutes an alternative measure of transmission intensity [6]. The conversion factor between FOI and SCR is equal to the proportion of infections which give rise to a detectable immune response, which may differ depending on the laboratory methods used. Serological data thus summarizes information on past exposure and should allow for measurement of the FOI using much smaller study populations, without the requirement for direct detection of parasites. Mathematically, the temporal change in the proportion of seropositives, *s*, can be described by the differential equation

(1) $$\frac{\mathrm{ds}}{\mathrm{dt}}=\lambda \left(1-s\right)-\mathrm{\rho s}\text{,}$$

With λ denoting the SCR, and *ρ* denoting the rate of seroreversion. The solution of the differential equation, assuming that at t = 0 the whole population is seronegative, indicates the proportion positive after time t as

(2) $$s\left(t\right)=\frac{\lambda }{\lambda +\rho }\left(1-e^{-\left(\lambda +\rho \right)t}\right)$$

This model, often called the “reversible catalytic model” [7] can be used to estimate SCR from the increase of seroprevalence with host age in cross-sectional data, considering that age is simply time since birth. Indeed, this method has been shown to yield good estimates of transmission intensity, which correlate well with EIR estimates from the same locations and capture geographic and temporal changes in FOI [4,8]. However, it is not suited to detecting single conversion events, partly because individual-level information is lost when converting continuous antibody titre measurements to a prevalence for each age group.

Serological cohort studies provide an interesting alternative: because the serological status of individuals is known at a minimum of two time points single infection events may be identified, allowing the measurement of low level transmission. Beyond measuring transmission intensity, analyses of serological cohort-data have the potential to improve the understanding of the dynamics of antibody responses.

However, serological data is very noisy: measured antibody levels may vary due to natural causes unrelated to malaria, such as stress or other infections, or due to small random errors in the processes of sample collection and laboratory analysis. As a consequence of this, using a fixed titre threshold to assign serological status is problematic in longitudinal data: some individuals may repeatedly pass the threshold merely due to small fluctuations in antibody levels, creating “false” conversion and reversion events. Consequently, a noise-robust statistical approach was used for the present analysis of a cohort of 160 Indonesian schoolchildren. A Hidden Markov Model (HMM) was fitted to the data in a two-step procedure: the titre distributions among negative and positive individuals, respectively, were estimated using a non-parametric Bayesian approach [9,10]. This allows assigning a probability of being seropositive to individuals, thus avoiding strict classification. Subsequently, the rates of transition between positive and negative states (the conversion and reversion rates) were estimated by Bayesian Markov Chain Monte Carlo (MCMC).

## Methods

### Study site and data collection

The present study was conducted in the district of Purworejo, Central Java Province, Indonesia, between December 2008 and June 2009. The area is characterized by low and seasonal malaria transmission, with higher intensity during the rainy season from October to March, and very low instensity during the dry season (April to August). A rolling cross-sectional study involving approximately 500 subjects indicated that the peak of monthly *P. falciparum* prevalence occurred during December 2008 (1.08%) while during the dry season almost no parasites were detected by microscopy (Supargiyono, unpublished). A total of 500 schoolchildren of age 10–11 was enrolled and tested for the presence of antibodies to *P. falciparum*. All children seropositive for at least one antigen at the December collection (n = 57), children with parasites during the cohort observation (n = 13) and a randomly chosen additional 90 seronegative individuals were selected for serological follow up, yielding a cohort of 160 individuals. Blood smears were taken weekly for 30 weeks for parasite microscopy. Parasitaemic individuals were always treated. Blood spots for serological analysis were collected monthly, from December 2008 to July 2009, on pre-labelled chromatographic filter paper (3 MM; Whatman, Maidstone, UK), and stored at −20°C until analysis. Ethical approval was received by the local institutional review board and written consent was received from the parents or guardians of the children.

### Microscopy and ELISA

Blood thick smears were stained with 5% Giemsa and a blood volume corresponding to at least 200 leucocytes was examined by a trained microscopist. Parasite density was calculated assuming 8000 leucocytes/μl. Blood spot samples collected on filter paper were cut in circles with a diameter of 3.5 mm (equivalent to 1.5 μl serum) and eluted with PBS-Tween (0.05%), as described previously [11], in preparation for analysis by enzyme-linked immunosorbent assay (ELISA). Antibody titres were measured using indirect ELISA as described in [12], using the *P. falciparum* merozoite surface protein 1_19_ (MSP-1_19_,) and *P. falciparum* apical membrane antigen 1 (AMA-1) recombinant proteins. Briefly, the PfMSP-1 and PfAMA-1 antigens were coated on plates at the concentration of 0.5 μg/mL in carbonate-bicarbonate coating buffer (pH 9.6) and incubated at 4°C overnight. After washing plates were blocked with 1% (w/v) skimmed milk solution for 3 hours. Samples were added in duplicate at a dilution of 1:1000 and a positive control pool of hyperimmune sera were added to each plate. After incubation overnight at 4°C, the plates were washed, horseradish-peroxydase-conjugated rabbit-anti-human IgG (DAKO, Roskilde, Denmark) was added, and plates were incubated for 3 hours. O-phenylenediamine was used as a substrate and the reactions were stopped by adding 25 μl 2 M H_2_SO_4_. Optical density was read at 450 nm.

### Data preparation

The raw OD data were converted to titre values by using a calibration curve generated by the positive control sera run on each plate. Only data of participants who were present at the six survey rounds from December to May were used for statistical analysis. Antibody data from the June survey was discarded due to a high proportion of missing values. This reduced the number of individuals in the data to 137, and the number of surveys to 6.

### Finite mixture models

Individuals are typically classified as seropositive if their antibody titre is higher than the mean of a control group plus two or three standard deviations [8,13]. However, this may yield biased estimates as some positive individuals may fall below this threshold while some negative individuals may exceed it. Subtle changes in actual antibody levels over time, perhaps due to co-infection with another pathogen, combined with small variation in the laboratory assays may be the reasons for such misclassification. The problem becomes particularly acute when considering longitudinal data (cohort studies): a single individual may - over the course of several observations - repeatedly pass the threshold even though the associated titre value was merely subject to noise and the underlying serological status essentially did not change. It is thus necessary for the analysis of longitudinal data to use a method of classification, which is robust against small changes in antibody titre.

“Finite Mixture Models” [14] provide a statistical framework for the analysis of data where the distribution of a continuous response variable, such as antibody titre, reflects the mixture of two or more classes of individuals, which have different but potentially overlapping responses. The overall distribution of antibody titres in the dataset (described by the probability density function (PDF) *f(x)*) is seen as a mixture of the titre distribution among negative individuals (with PDF *g*_*–*_*(x)*) and the distribution among positives (with PDF *g*_*+*_*(x)*). With the mixing parameter *π* – the seroprevalence – the distribution of titres can be written as

(3) $$f\left(x\right)=\left(1-\pi \right)g_{-}\left(x\right)+\pi g_{+}\left(x\right)\text{.}$$

In words: the PDF of the overall titre distribution is a weighted average of the titre distributions in the positive and negative classes.

### Mixture decomposition

The method of [9] was used to estimate the mixture parameters. It requires a statistical sample of titre values from an unexposed control group. The titre distribution among controls is compared to the distribution in the field data, which is known to comprise a mixture of positive and negative individuals. The most likely values for the titre distribution among positives, negatives, and the mixing ratio (seroprevalence) can then be determined.

An implementation for use with WINBUGS software [15] and documentation can be found at [16]. The approach is non-parametric, meaning that no particular shapes of the distributions *g*_*–*_*(x)* and *g*_*+*_*(x)* have to be assumed. Instead, the titre values are grouped into ordered categories, and parameter estimation is subject to the two constraints that i) the odds of being positive, *g*_*+*_*(x)/g*_*-*_*(x),* are strictly increasing with titre, and ii) that there are no positives in the category with the lowest titre values. Model fitting by Markov Chain Monte Carlo (MCMC) yields estimates of the overall seroprevalence *π* as well as the following parameters for every titre category *i*: the proportion of positives in each titre category, *π*_*i*_, and the proportion of seronegatives, *θ*_*i*_, and seropositives, *ϕ*_*i*_, respectively, which have a titre value in category *i*.

For the present analysis, titre ranges were defined according to the following criteria: the first category includes titres up to 10 arbitrary units (AU) in order to justify the required assumption that the first category contains no positives; the range of subsequent categories was required to be no less than 30 AU and to contain a minimum of 10 data points. The titre values of 40 unexposed Javanese individuals from Yogyakarta, Java, were used as negative control group. The medians of the posterior samples were used as point estimates for all parameters. These are shown in Tables 1 (PfAMA-1) and 2 (PfMSP-1), with the 95% Bayesian credible intervals. The re-normalized probabilities *π*_*i*_ and *ϕ*_*i*_ were then divided by the size of the corresponding titre category in order to obtain probability densities *g*_*-*_*(x)* and *g*_*+*_*(x)*.

**Table 1:** Mixture decomposition (AMA-1)

** i**	** Titre range**	** π**_**i**_	** φ**_**i**_	** θ**_**i**_
1	-∞ < t < 10	0 (by definition)	0 (by definition)	0.247 (CI: 0.219 - 0.276)
2	10 < t < 40	3.06E-5 (CI: 1.67E-8 – 5.52E-3)	8.52E-4 (CI: 7.25E-7 – 0.0755)	0.382 (CI: 0.349 – 0.414)
3	40 < t < 70	8.26E-5 (CI: 5.59E-8 – 0.0108)	1.02E-3 (CI: 1.17E-6 – 0.0621)	1.68E-1 (CI: 1.44E-1 – 0.194)
4	70 < t < 100	2.21E-4 (CI: 2.12E-7 – 0.0208)	9.88E-4 (CI: 1.58E-6 – 0.0415)	0.0600 (CI: 0.0454 – 0.0772)
5	100 < t < 130	6.03E-4 (CI: 8.08E-7 – 0.0390)	1.59E-3 (CI: 3.70E-6 – 0.0444)	0.0356 (CI: 0.0245 – 0.0495)
6	130 < t < 160	1.63E-3 (CI: 3.01E-6 – 0.0706)	3.18E-3 (CI: 1.05E-5 – 0.0593)	0.0263 (CI: 0.0170 –0.0383)
7	160 < t < 190	4.38E-3 (CI: 1.17E-5 – 0.125)	5.92E-3 (CI: 3.02E-5 – 0.0741)	0.0182 (CI: 0.0107 – 0.0285)
8	190 < t < 228	0.0115 (CI: 5.003E-5 – 0.209)	9.77E-3 (CI: 7.91E-5 – 0.0831)	0.0113 (CI: 5.61E-3 – 0.0198)
9	228 < t < 258	0.0300 (CI: 1.97E-4 – 0.332)	0.0281 (CI: 3.87E-4 – 0.156)	0.0124 (CI: 6.43E-3–0.0213)
10	258 < t < 541	0.0748 (CI: 8.63E-4 – 0.485)	0.0657 (CI: 1.70E-3 – 0.244)	0.0113 (CI: 5.57E-3 – 0.0198)
11	541 < t < 710	0.174 (CI: 3.68E-3 – 0.644)	0.163 (CI: 0.0100 – 0.418)	0.0112 (CI: 5.60E-3 – 0.0197)
12	710 < t < 1547	0.352 (CI: 0.0156 – 0.801)	0.384 (CI: 0.0838 – 0.794)	0.0113 (CI: 5.60E-3 – 0.0198)
13	1547 < t < 1730	0.577 (CI: 0.0415 – 0.942)	0.161 (CI: 0.0187 – 0.818)	1.95E-3 (CI: 2.88E-4 – 6.45E-3)

Medians and Bayesian Credible Intervals (CI, 2.5^th^ to 97.5^th^ percentile) of the estimated seroprevalence π_i_ in each titre range i and the probabilities φ_i_ and θ_i_ that a seropositive or -negative person, respectively, has an AMA-1 titre in range i. The overall seroprevalence π was 0.0169 (CI: 6.24E-4 – 0.0888). The titres of 40 unexposed individuals living in Yogyakarta served as negative control group.

### Estimation of conversion and reversion rates using Hidden Markov Models

Hidden Markov Models (HMM´s) are a popular, flexible class of statistical time-series models, which can be seen as a generalization of finite mixture models, as described above, with the mixing ratio changing over time [14]. In the present context, the sequence of observed titres x = (*x*_*1*_*…x*_*n*_), obtained from one subject at surveys 1..*n*, is seen as the result of a “hidden” (unobserved) sequence of states h = (*h*_1_…*h*_*n*_), with *h*_*t*_ corresponding to the serological state at survey t. The titre *x*_*t*_ at time t is distributed according to a particular PDF *g*_*ht*_*(x)*, conditional on the hidden state *h*_*t*_. Transitions between hidden states occur with probabilities *q(h*_*t-1*_*, h*_*t*_*)*. This implies a major simplifying assumption of HMM's: the stochastic process governing state transitions is without memory, and only the immediately preceding hidden state influences transitions. Further, the progression through antibody levels after seroconversion is not explicitly modelled, but simply summarized in the titre distribution among positives.

**Figure 1:**
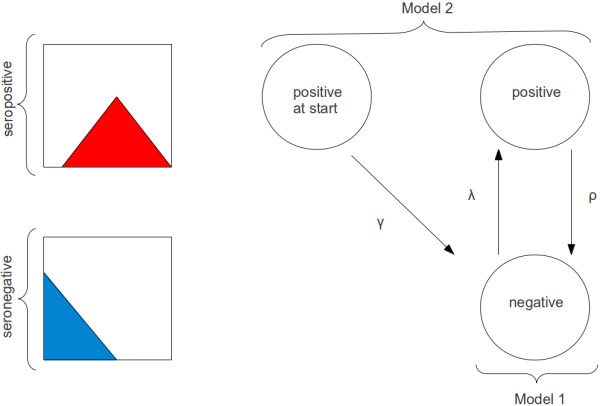
**Models for the transitions between serological states.** Two models for the transition of individuals from positive (top row) to negative (bottom row) serological states over the course of the study: Model 1 assumes one positive and one negative state, with conversion rate λ and reversion rate ρ. Model 2 treats individuals separately which are already seropositive at the start of the study. Those revert at a rate γ, which may be different from ρ. Titre distributions in positive and negative individuals are schematically indicated on the left.

Two distinct continuous-time models for the transition of individuals between hidden states were compared (Figure 1). Model 1 corresponds exactly to the reversible catalytic model described above, with a negative and a positive serological state, and seroconversion and -reversion rates λ and ρ, respectively. The distribution of antibody titres in either state corresponds to the estimates of the negative and positive component, respectively, as obtained from the mixture separation described above. Model 2 introduces an additional positive state, “positive at start”, with a separate reversion rate γ. This parameter is not of direct interest, but serves the purpose of “absorbing” the transmission history of the study population: Model 1 ignores that subjects who converted long before the study started may revert at a different rate – faster or slower. An estimate of the reversion rate, which is the inverse of the mean duration of seropositivity and, therefore, itself of interest, would thus be biased. For PfAMA-1 and PfMSP-1 antibody data separately, the two models were compared with respect to goodness of fit by means of the deviance information criterion (DIC). Smaller values of DIC indicate a better fit to the data [17].

### Likelihood computations

The set of possible hidden states, denoted by *S*, comprises a negative and a positive state in the case of Model 1 (*S* = {1, 2}), and one negative and two positive states in the case of Model 2 (*S* = {1, 2, 3}), as illustrated in Figure 1. The intensity matrix I, which indicates the flow from each state i (rows) to each other state j (columns), takes the form

$$I_{1}=\left[\begin{array}{c} -\lambda \;\lambda \\ \rho \;-\rho \end{array},\;\right]$$

for Model 1, and

$$I_{2}=\left[\begin{array}{c} -\gamma \;\gamma \;0\\ \;0\;-\lambda \;\lambda \\ \;0\;\rho \;-\rho \end{array},\;\right]$$

for Model 2. For each intensity matrix I, the corresponding matrix Q, containing the discrete-time transition probabilities *q(i, j)* from state i (rows) to state j (columns) during one survey interval, can then be obtained as Q = e^I^, which is equivalent to integration of the system of ordinary differential equations defined by I over one time unit.

The likelihood *p(x*_*t*_*|h*_*t*_*, g*_*-*_*, g*_*+*_*)* of a single titre observation in an individual equals *g*_*-*_*(xt)* if the hidden state *h*_*t*_ is negative, and *g*_*+*_*(x*_*t*_*)* if it is positive. However, since the hidden state sequence is unknown, the overall likelihood is computed as a weighted average of likelihoods across all possible hidden sequences [14]. Likelihoods are weighed by the probability of each hidden sequence occurring, which can be obtained from the probability *β(h*_*1*_*)* of being in a particular state *h*_*1*_ at the first survey, and the transition probabilities between successive states, *q(h*_*t-1*_*, h*_*t*_*)*. In the present analysis, *β(h*_*1*_*)* was estimated as a free parameter separately for each individual. The marginal likelihood of the data given the state transition rates can thus for one individual be written as follows (details in notation omitted):

(4) $$\begin{array}{l} p\left(x\right)=\sum_{h\in S^{n}}p\left(x,h\right)\\ \;=\sum_{h\in S^{n}}\beta \left(h_{1}\right)p\left(x_{1}|h_{1}\right)\prod_{t=2}^{n}q\left({h_{t}}_{-1},h_{t}\right)p\left(x_{t}|h_{t}\right), \end{array}$$

and *Σ*_*i*_*log(p(x*_*i*_*)* of the likelihoods for every person i yields the overall log-likelihood for the data on one particular antibody. Parameter estimates and Bayesian Credible Intervals were obtained for each antibody type separately by Bayesian Markov-Chain-Monte-Carlo (MCMC) simulation, carried out using the JAGS program [18] in conjunction with the R statistical software [19]. For all parameters, non-informative uniform prior distributions were chosen.

### Finding individuals which seroconverted during the study

The individuals which most likely seroconverted during the study period were identified by calculating for every survey pair the probability that seroconversion occurred based on the combined titre changes of both antibodies, PfAMA-1 and PfMSP-1. Given the titres at the two surveys $x_{1}^{\mathrm{AMA}1},x_{2}^{\mathrm{AMA}1},x_{1}^{\mathrm{MSP}1},x_{2}^{\mathrm{MSP}1}$, summarized as X, and the PDFs of the corresponding mixture components, $g_{-}^{\mathrm{AMA}1},g_{+}^{\mathrm{AMA}1},g_{-}^{\mathrm{MSP}1},g_{+}^{\mathrm{MSP}1}$, summarized as **G**, this probability can be calculated as follows: let

$$\begin{array}{l} g_{h_{1}}^{\mathrm{AMA}1}\left(x_{1}^{\mathrm{AMA}1}\right)g_{h_{2}}^{\mathrm{AMA}1}\left(x_{2}^{\mathrm{AMA}1}\right)g_{h_{1}}^{\mathrm{MSP}1}\left(x_{1}^{\mathrm{MSP}1}\right)g_{h_{2}}^{\mathrm{MSP}1}\left(x_{2}^{\mathrm{MSP}1}\right)\\ \;=P_{h_{1}h_{2}}\left(X|G\right) \end{array}$$

be the likelihood of the data given one of the four possible hidden state sequences, and assume that all of these are equally likely before considering the data (i.e. have prior probability 1/4), then the probability of seroconversion is

(5) $$\frac{p_{-+}\left(X|G\right)}{p_{--}\left(X|G\right)+p_{-+}\left(X|G\right)+p_{+-}\left(X|G\right)+p_{++}\left(X|G\right)}=\mathrm{Pr}\left(h_{1}=-,h_{2}=+\right)$$

via Bayes' Theorem. This probability was calculated for every pair of observations in every individual in order to find those with the highest probability of conversion.

All analyses were performed using the R statistical software package [19], in conjunction with JAGS [18] for Bayesian analyses.

## Results

Of the 137 study participants, the number of microscopy-positive individuals at rounds 1 through 4 (Dec-Mar) was 7, 3, 2, and 1, respectively. The corresponding point prevalences are 0.05, 0.02, 0.01 and 0.007. No positives were found at rounds 5 and 6.

**Figure 2:**
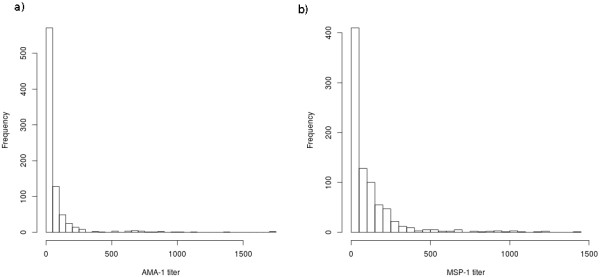
**Distribution of AMA-1 and MSP-2 titres.** The titre distribution of anti AMA-1 (**a**) and anti MSP-1 (**b**) antibodies in the study population, all survey rounds pooled.

The median antibody titres and corresponding inter-quartile ranges (IQR) per round were 36 (15–64), 31 (16–72), 38 (19–64), 24 (12–66), 20 (8–55) and 12 (5–41) for AMA-1, and 54 (19–165), 37 (7–121), 45 (15–143), 65 (18–136), 49 (17–139) and 48 (10–118) for MSP-1. The titre distributions are shown in Figure 2. A Kruskal-Wallis test indicated that AMA-1 titres differed between time points (p < 0.0001), while MSP-1 titres did not differ (p = 0.14).

**Figure 3:**
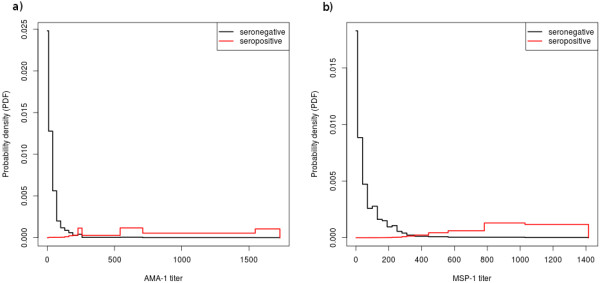
**Results of the mixture decomposition.** Graphical representation of the estimated probability densities of the titre distributions in seronegative and -positive individuals, respectively, for both AMA-1 (**a**) and MSP-1 antibodies (**b**). An average of the PDFs, weighted by relative abundance of positive and negative individuals, approximately yields the PDF of the titre data.

The nonparametric Bayesian mixture decompositions estimated an overall seroprevalence in the dataset of 0.0169 (CI: 6.24E-4 – 0.0888) for AMA-1 and 0.0168 CI (6.27E-4 – 0.0863) for MSP-1. Outputs of the mixture separation according to the method of [9] are given in Table 1 (AMA-1) Table 2 (MSP-1), and Figure 3. Median titre and IQR among negative controls was 11.6 (4.32 – 16.6) for AMA-1 and 30.3 (11.6 – 80.9) for MSP-1.

**Table 2:** Mixture decomposition (MSP-1)

** i**	** titre range**	** π**_**i**_	** φ**_**i**_	** θ**_**i**_
1	-∞ < t < 10	0 (by definition)	0 (by definition)	0.932 (CI: 0.157 – 0.209)
2	10 < t < 40	3.94E-7 (CI: 6.62E-11 – 2.31E-4)	7.43E-6 (CI: 1.93E-9 – 2.67E-3)	0.264 (CI: 0.236 – 0.294)
3	40 < t < 70	1.05E-6 (CI: 2.29E-10 – 4.76E-4)	1.08E-5 (CI: 3.55E-9 – 2.91E-3)	0.1413 (CI: 0.119 – 0.166)
4	70 < t < 100	2.82E-6 (CI: 8.20E-10 – 9.84E-4)	1.59E-5 (CI: 7.16E-9 – 3.21E-3)	0.0774 (CI: 0.0608 – 0.0965)
5	100 < t < 130	7.57E-6 (CI: 2.73E-9 – 2.04E-3)	4.61E-5 (CI: 2.61E-8 – 6.99E-3)	0.0831 (CI: 0.0661 – 0.103)
6	130 < t < 160	2.06E-5 (CI: 1.00E-8 – 4.09E-3)	7.23E-5 (CI: 5.58E-8 – 7.86E-3)	0.0484 (CI: 0.0354 – 0.0640)
7	160 < t < 190	5.57E-5 (CI: 3.76E-8 – 8.28E-3)	1.82E-4 (CI: 1.94E-7 – 0.0146)	0.0449 (CI: 0.0325 – 0.0599)
8	190 < t < 220	1.51E-4 (CI: 1.39E-7 – 0.0159)	3.13E-4 (CI: 4.49E-7 – 0.0175)	2.87E-2 (CI: 1.89E-2 – 0.0412)
9	220 < t < 250	4.06E-4 (CI: 4.99E-7 – 0.0310)	9.43E-4 (CI: 1.85E-6 – 0.0362)	3.20E-2 (CI: 2.17E-2 – 0.0452)
10	250 < t < 280	1.10E-3 (CI: 1.85E-6 – 0.0580)	1.35E-3 (CI: 3.79E-6 – 0.0360)	1.70E-2 (CI: 9.79E-3 – 0.0272)
11	280 < t < 310	2.96E-3 (CI: 6.88E-6 – 0.104)	2.66E-3 (CI: 1.13E-5 – 0.0465)	1.24E-2 (CI: 6.42E-3 – 0.0213)
12	310 < t < 358	7.92E-3 (CI: 2.73E-5 – 0.180)	6.51E-3 (CI: 4.12E-5 – 0.0733)	1.12E-2 (CI: 5.59E-3 – 0.0198)
13	358 < t < 442	0.0210 (CI: 1.13E-4 – 0.291)	0.0176 (CI: 1.87E-4 – 0.124)	0.0112 (CI: 5.59E-3 – 0.0197)
14	442 < t < 561	0.0539 (CI: 4.93E-4 – 0.431)	0.0467 (CI: 8.72E-4 – 0.206)	1.12E-2 (CI: 5.56E-3 – 0.0198)
15	561 < t < 781	0.129 (CI: 2.20E-3 – 0.587)	0.120 (CI: 4.82E-3 – 0.343)	0.0112 (CI: 5.55E-3 – 0.0197)
16	781 < t < 1029	0.278 (CI: 9.45E-3 – 0.741)	0.283 (CI: 0.0351E-2 – 0.610)	0.0113 (CI: 5.57E-3 – 0.0198)
17	1029 < t < 1417	0.500 (CI: 0.0323 – 0.888)	0.399 (CI: 0.122 – 0.932)	6.60E-3 (CI: 2.58E-3 – 0.0136)

Medians and Bayesian Credible Intervals (CI, 2.5^th^ to 97.5^th^ percentile) of the estimated seroprevalence π_i_ in each titre range i and the probabilities φ_i_ and θ_i_ that a seronegative or -positive person, respectively, has an MSP-1 titre in range i. The overall seroprevalence π was 0.0168 CI (6.27E-4 – 0.0863). The titres of 40 unexposed individuals living in Yogyakarta served as negative control group.

Of the Hidden Markov Models, Model 1 fitted both AMA-1 as well as MSP-1- data better than Model 2, as indicated by lower values of DIC (Table 3). Estimates of the seroconversion rate λ by Model 1 are 0.0157 (CI 5.78E-4 – 0.0827) for AMA-1, and 0.0872 (CI 0.0235 – 0.210) for MSP-1. The corresponding rates of seroreversion,*ρ*, were 0.553 (CI 0.0404 – 1.71) and 2.26 (CI 0.892 – 4.34), respectively. Parameter estimates of Model 1 were not sensitive to changes in the uniform prior distributions. In Model 2, only the reversion rate ρ was not identifiable and showed strong dependence on the prior distribution when fitted to the AMA-1 data.

**Table 3:** Parameter estimates

**AMA-1**				
**Model**	**λ**	**ρ**	**γ**	**DIC**
1	0.0157 (5.78E-4 – 0.0827)	0.553 (0.0404 – 1.71)	n.a.	2964
2	0.0187 (7.09E-4 – 0.103)	5.58 (0.308 – 9.80)	0.541 (0.0389 – 1.68)	2968
**MSP-1**				
**Model**	**λ**	**ρ**	**γ**	**DIC**
1	0.0872 (0.0235 – 0.210)	2.26 (0.892 – 4.34)	n.a.	3829
2	0.0875 (0.0235 – 0.209)	0.926 (0.0328 – 5.49)	3.12 (1.23 – 6.52)	3833

The seroconversion rate λ and reversion rates *ρ* and *γ* as estimated by Models 1 and 2 (person^-1^ year^-1^) are shown with 95% Bayesian credible intervals. Models were fitted to AMA-1 and MSP-1 data separately. Lower values of Deviance Information Criterion (DIC) indicate a better fit to the data.

**Figure 4:**
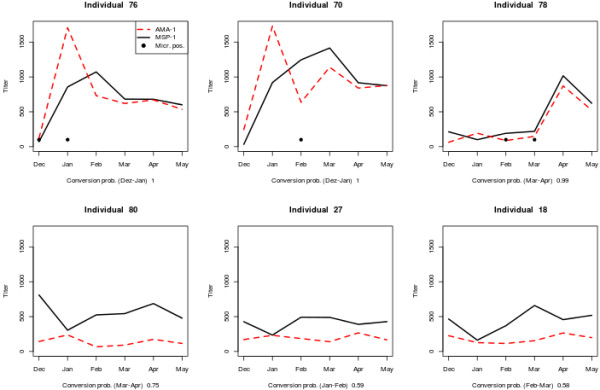
**Seroconverting individuals.** Those individuals most likely experiencing seroconversion during the study period are displayed in descending order of conversion probability, as determined from pairs of surveys. Only individuals 76, 70 and 78 appear to have converted with near certainty, and do not revert within the study period. Black dots indicate the presence of parasites in blood slides. Since parasitaemic individuals were always treated, those may represent re-infections.

**Figure 5:**
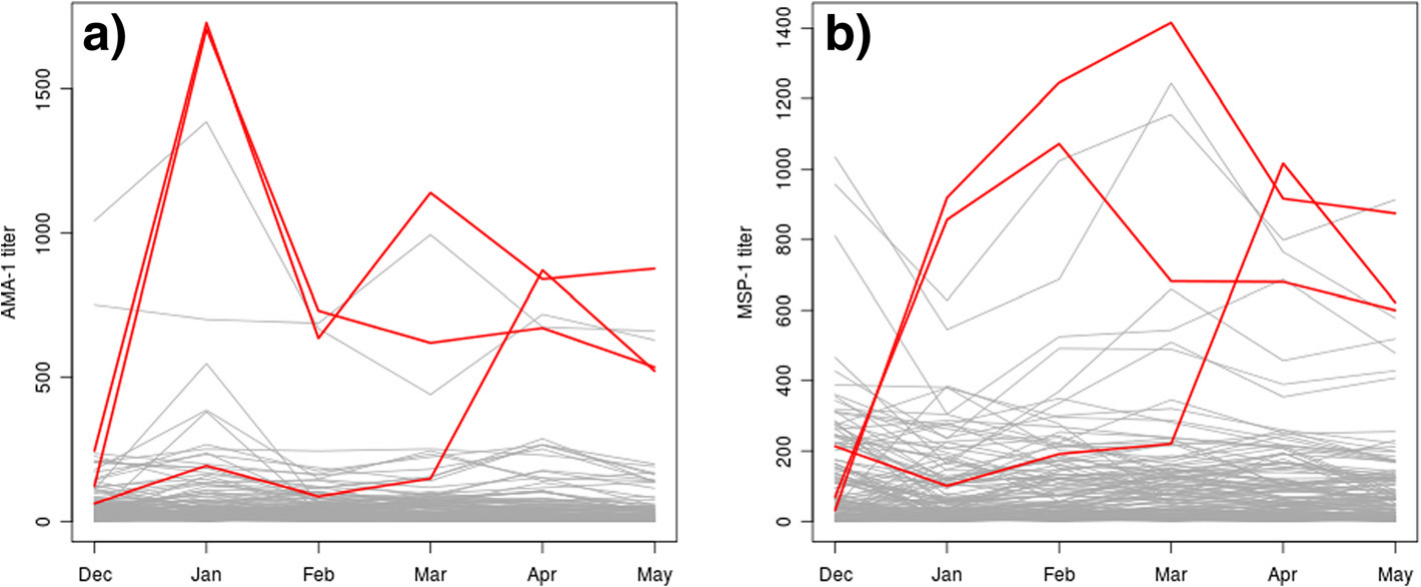
**Titre time series.** Antibody titre time series of the three individuals which likely seroconverted during the study (red) are shown against the background of the whole study populations; separately for antibodies against AMA-1 (**a**) and MSP-1 (**b**).

Individuals 76, 70, and 78 experienced seroconversion with near certainty (conversion probability > 95%). The titre time series of these are shown separately for each individual in Figure 4 (top row), and against the background of the entire study population in Figure 5. In addition, individuals 80, 27, 18 and 30 may have converted (conversion probability > 50%), but no clear conversion event is visible by eye. The first three of these are shown in Figure 4 (bottom row). For comparison, a positivity threshold was defined using the log-transformed titre values of the control group: a log-titre larger than two (three) standard deviations from the mean was considered positive. This corresponded to a titre threshold at 252.8 (731.1) for MSP-1, and at 79.9 (243.5) for AMA-1. By this method, 14 (5) conversions were counted in the MSP-1 data, and 32 (13) conversions using AMA-1. The count of reversions was 24 (6) for MSP-1, and 40 (11) for AMA-1.

## Discussion

The significant decrease of (only) anti AMA-1 antibody titres and the decreasing number of parasite-positive individuals are consistent with the seasonal drop in transmission intensity in the study area. A rolling cross-sectional study in the same area, where the peak of transmission occurred in December, found no parasites during the dry season from April to August (Supargiyono, unpublished). SCR estimates by the best-fitting HMM indicate very small levels of *P. falciparum* transmission during the study period, with estimates based on AMA-1 considerably lower than those based on MSP-1: 0.0157 person^-1^ year^-1^ for AMA-1 and 0.0872 person^-1^ year^-1^ for MSP-1. These SCR estimates are based on the proportion of seronegative individuals who convert and become seropositive per year. Although infections in positive individuals are “not counted” by the model, the rate estimates are unbiased since the denominator contains only the number of persons at risk of converting (the seronegatives). The rate estimates imply that one should expect to find between 0.896 and 4.98 infection events in the present dataset, where 137 individuals were followed for 5 months. This is compatible with the findings from the pairwise analysis which found three individuals who almost certainly experienced infection (conversion probability > 95%), and an additional four which may have done so (conversion probability > 50%).

All individuals which converted with probability > 95% also tested parasite positive either at the same survey (ind. 76), before (ind. 78) or after (ind. 70) the antibody response (Figure 4). Because the sensitivity of microscopy is far from perfect [20] it is difficult to determine the exact time point of infection from microscopy data. This might explain antibody responses which precede microscopic detection, but a delayed antibody response may indicate that not all hosts respond immediately and/or against all antigens of a parasite, an idea that appears plausible when looking at the titres of individuals that ever tested positive by microscopy (Additional file 1). Future analyses might thus consider using a larger number of antigens simultaneously, and extend the statistical methods accordingly.

The consistency of model-based SCR estimates with the number of conversion events identified by pairwise comparison of titres demonstrates that the HMM approach is both robust against noise in titre measurements as well as highly sensitive at detecting low levels of serological incidence. It may thus be used to measure the FOI at very low levels of transmission, which may be encountered in a near-elimination scenario or when preventing re-introduction of the disease after successful elimination. Once malaria has completely disappeared or is very rare, it is ethically problematic to collect large numbers of blood samples for the purpose of measuring transmission intensity [2]. However, antibody titres can also be measured non-invasively from saliva samples, which would allow large-scale screening of affected populations [21]. Serological cohort-data from saliva samples in conjunction with the statistical analysis approach presented here may thus represent a formidable tool for post-elimination surveillance.

In addition to the rate of conversion, the HMM's yield an estimate of the rate of sero-reversion which is the inverse average duration of seropositivity. The duration of seropositivity is likely to be different in children compared to adults due to physiological changes with age; in addition, it may differ between antigens, and is expected to increase with cumulative exposure. The duration of seropositivity, and how it is affected by the above factors, is of major interest for the planning of studies which use antibodies for epidemiological monitoring and for choosing the best-suited antigens. In addition, the change in duration of antibody responses in response to cumulative exposure has the potential to yield further insight into the acquisition of immunological memory against malaria. The present analysis attempted to obtain estimates of the seroreversion rate which are unbiased by the exposure history of the study population; a central assumption of a HMM is that the probability of reversion at any moment is independent of how long the individual has already been positive. Since this is not strictly true in the biological counterpart, Model 2 was devised to “absorb” the bias on reversion rate estimates introduced by individuals already positive at the start of the study. However, Model 1 fitted the data better, which suggests that the present dataset does not contain enough information to measure reversion rates strictly from individuals which converted (and reverted) during the study. This is in line with the observation that none of the three clearly converting individuals (Figure 5, top row) appears to revert during the study. The estimates of *ρ* obtained from Model 1 thus provide only limited information on the actual duration of seropositivity. In principle, however, the present approach allows measurement of the duration of antibody responses, but cohort data are required where enough individuals both convert and revert during the study period. Ideally, this requires larger datasets where transmission intensity is somewhat higher but still low enough such that multiple concurrent infections per person are rare. Effects of individual inoculations on antibody responses would then remain distinguishable.

The advantages of the statistical methods used in the present analysis, compared to threshold-based methods, are mostly in their robustness towards noise in the titre measurements as well as in their comparatively solid theoretical foundation. The pairwise analysis yields a probability measure indicating whether seroconversion has happened (with values close to 1.0 equivalent to near certainty), based on relatively few assumptions. The HMMs propagate uncertainties concerning serological status into the conversion- and reversion rate estimates, in form of wider credible intervals. The number of seroconversions and -reversions recorded using the cutoff method depended strongly on the (arbitrary) numerical value of the positivity threshold; generally, more conversions/reversions were counted. It appears plausible that random fluctuations in the titre measurements created “false” conversion and reversion events, which renders SCR estimates obtained in this manner rather unreliable. The approaches introduced in this article, in contrast, are more robust because they weigh large titre changes more than small ones, thereby making better use of the information in the data.

**Figure 6:**
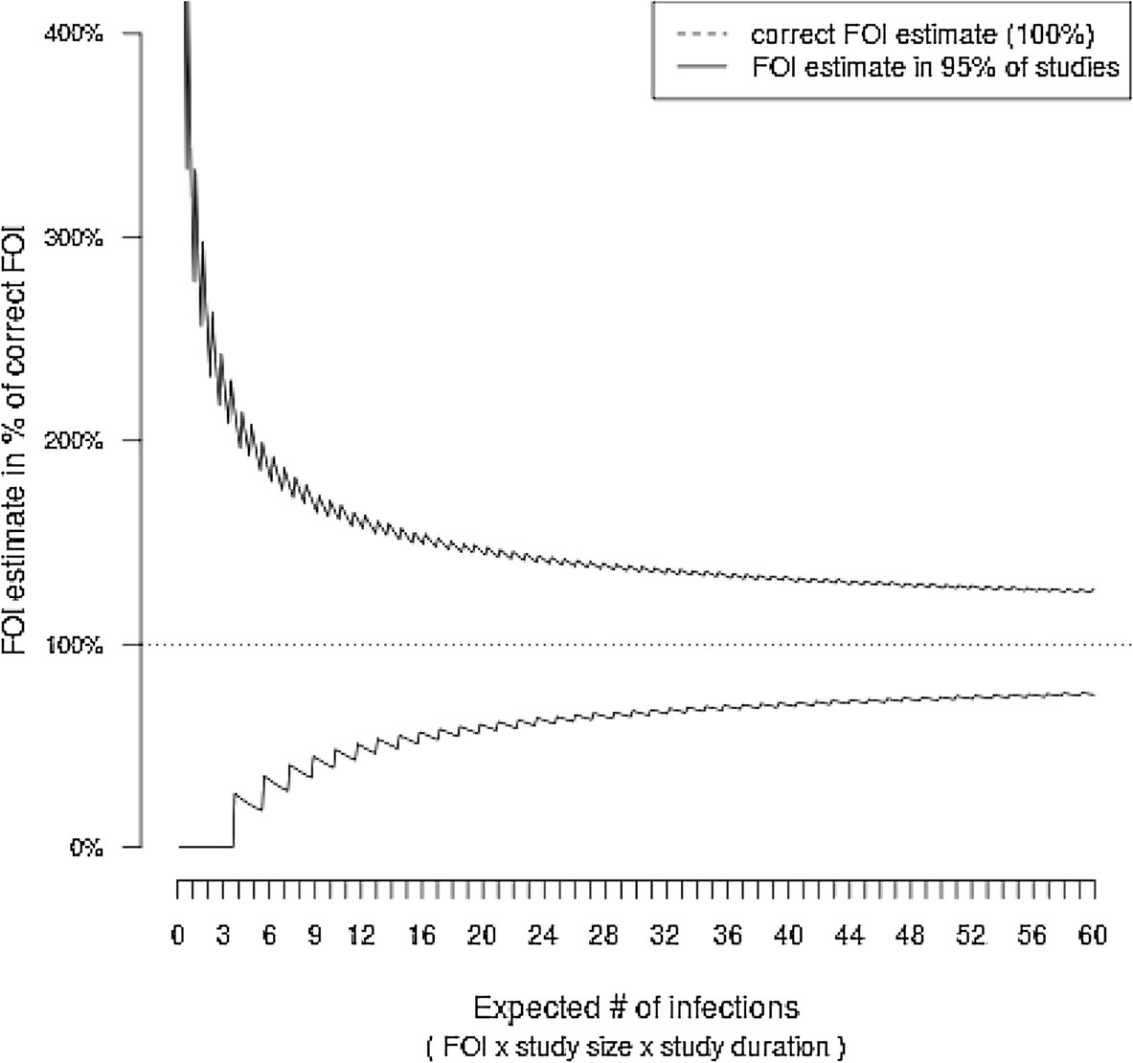
**Theoretical limits for measuring transmission intensity in cohort studies.** Measuring the force of infection (FOI) in a cohort study by detecting infection events is subject to the theoretical limitations governing count data: under idealizing assumptions the number of infections in a study is Poisson-distributed with expectation equal to FOI x the number of study subjects x study duration. This introduces uncertainty into FOI estimates. An example: in order to measure a FOI with ca. ± 25% accuracy, on average 50 infections need to happen during a study. At a FOI of 0.1, this can be achieved by following 500 individuals for 1 year, or 250 individuals for 2 years, etc.

The uncertainty in the present SCR estimates is considerable, in fact similar in magnitude as the estimates themselves. This is not only due to the uncertainty in classifying individuals as positive or negative, but because sample size and duration of follow up are rather small and do not allow for more precise measurement of such low-intensity transmission, even if a perfect method of identifying infections were available. Figure 6 illustrates the relationship between the theoretical limits of measuring transmission intensity and the dimensions of a study. The underlying model assumes that a non-heterogeneous force of infection is acting on a study population, and that a perfect method for counting infections is available. The number of infection observed is then Poisson-distributed. This introduces uncertainty into the corresponding rate estimates. For the present study with three detected infections, estimates from 0% to 200% of the true value are to be expected, even with optimal methods. The scarcity of conversion events in this data also precludes addressing seasonality of transmission.

## Conclusions

Serological cohort studies are an efficient means of obtaining information on the FOI in areas of very low transmission and single conversion events may be detected. The statistical methods presented here suggest that this approach could be a useful adjunct measure to existing measures of transmission such as clinic based incidence rates especially if targeted to easy access groups. More serological data are required from cohorts resident at different endemicities to further validate the approach.

## Competing interests

The authors declare that they have no competing interests.

## Authors’ contributions

MTB performed the statistical analyses and wrote the manuscript; SS conducted the study, coordinated the field- and laboratory-based analyses and assisted in writing the manuscript; MAW coordinated blood sample collection, microscopic blood slide examination and laboratory analyses; DN and ANW performed the ELISA assays; NFL assisted with design and conducting of the study; WAH assisted with study design and revised the manuscript; JC assisted in laboratory analyses, processed the raw data and performed initial statistical analyses; SS, CJD, WAH designed and coordinated the study and revised the manuscript. All authors have read and approved the final manuscript.

## Supplementary Material

Additional file 1 A plot of the data from all individuals with at least one positive microscopy result. Click here for file
