# Supplementary figures and images for: Measurement of Plasmodium falciparum transmission intensity using serological cohort data from Indonesian schoolchildren
Source: Malar J. 2013 Jan 17;12:21. doi: 10.1186/1475-2875-12-21 (PMC3605132; doi:10.1186/1475-2875-12-21)

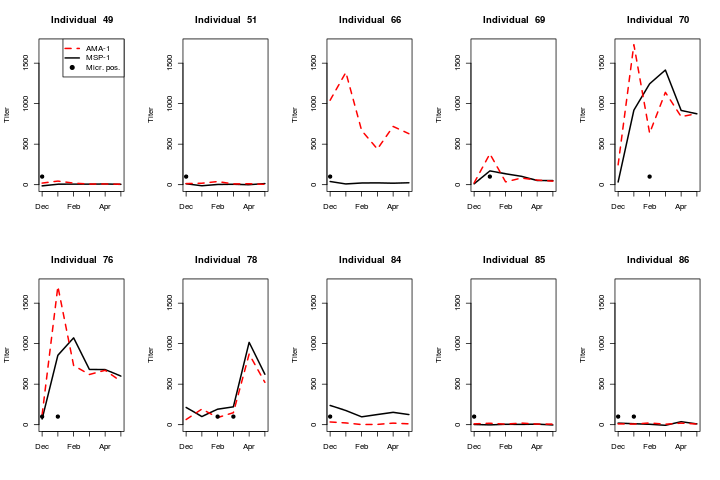

Supplement: Additional file 1 — A plot of the data from all individuals with at least one positive microscopy result. [file 1475-2875-12-21-S1.png]
